# Trauma exposure and alcohol use disorder among prisoners in Jimma Zone correctional institution, Southwest Ethiopia: a cross-sectional study

**DOI:** 10.1186/s13104-019-4796-9

**Published:** 2019-11-19

**Authors:** Yimenu Yitayih, Matiwos Soboka, Elias Tesfaye, Mubarek Abera, Almaz Mamaru, Kristina Adorjan

**Affiliations:** 1 Department of Psychiatry, Faculty of Medical Sciences, Jimma University, Jimma, Ethiopia; 2 Institute of Psychiatric Phenomics and Genomics (IPPG), Medical Center of the University of Munich, Munich, Germany; 3 Departments of Psychiatry and Psychotherapy, Medical Center of the University of Munich, Munich, Germany; 4 Center for International Health, Ludwig-Maximilians-Universität, Munich, Germany

**Keywords:** Crime, Prisoners, Trauma, Alcohol use disorder, Khat abuse, Tobacco dependence

## Abstract

**Objective:**

Trauma exposure and alcohol use are closely related, and large proportion of trauma-exposed individuals use alcohol. The data presented in this paper were obtained as part of a study on substance use disorder and associated factors among prisoners in the correctional institution in Jimma, Southwest Ethiopia. Therefore, in this study we examined comorbidity of traumatic life experiences and alcohol use disorder in inmates of correctional institution in Jimma, Southwest Ethiopia.

**Results:**

The overall prevalence of lifetime alcohol use disorder was 40.1%, and the prevalence of alcohol use disorder among prisoners with lifetime trauma exposure was 44.0%. Participants with multiple trauma exposures had 2.5-fold higher odds of association for alcohol use disorder than their counterparts (AOR = 2.47 [1.23–4.94]). Living in urban areas (AOR = 4.86 [2.38–9.94]), presence of psychopathy (AOR = 3.33 [1.25–8.86]), khat abuse (AOR = 7.39 [3.99–13.68]), and nicotine dependence (AOR = 2.49 [1.16–5.34]) were also positively associated with alcohol use disorder. The prevalence of alcohol use disorder was higher among prisoners with lifetime trauma exposure. Also, this study indicates that prisoners with multiple trauma exposures had higher odds of association for alcohol use disorder than those with no trauma exposure. A public health intervention targeting survivors of traumatic experiences needs to be designed and implemented.

## Introduction

Traumatic events are extremely prevalent; they are related to adverse mental health and have social and economic consequences [1]. The prevalence of exposure to traumatic events according to DSM-5 criteria is 89.7% [2]. A national trauma comorbidity report found that 60% of men and 50% of women experience trauma in their lifetime [3]. Such events are an almost universal experience among prisoners [1]. One study found trauma exposure rates ranging from 62.4 to 87.0% among incarcerated adult males [4]. Another study showed that 22% to 43% of patients with posttraumatic stress disorder (PTSD) develop a substance use disorder over the course of their lives [5]. Recent research has also identified PTSD to be a risk factor for substance use relapse and worse treatment outcome [6, 7].

Studies have identified several potential mechanisms to explain the relationship between trauma exposure and AUDs. For example, Boys and Marsden defined the self-medication hypothesis as a specific type of functional substance use [8]. In the context of substance use, the self-medication hypothesis suggests that people use alcohol to suppress or reduce the burden of posttraumatic symptoms [9]. In addition, alcoholic patients with a high trauma load often functionally consume alcohol to cope with sleep issues, depression, or intrusive memories [10]. According to the behavioral sensitization paradigm, repeated administration of substances or exposure to stress can cause sensitization of dopamine neurons, resulting in a higher dopamine release in response to subsequent stress and substance abuse [11].

The high-risk hypothesis claims that people with a substance disorder have a higher likelihood of developing PTSD because their risky lifestyle is likely to lead to adverse, stressful, and negative life experiences, which can then lead to PTSD [12, 13]. The vulnerability may be influenced by a lack of coping strategies or neurochemical brain changes caused by substance use [14].

The data presented in this paper were obtained as part of a study on substance use disorder and associated factors among prisoners in the correctional institution in Jimma, Southwest Ethiopia [15]. As noted, previous research has addressed the association between trauma exposure and substance use disorders in general [15]; however, to our knowledge the potential association of traumatic life events with problematic alcohol use has not been examined among prison populations in Ethiopia. Therefore, this study aimed to assess trauma exposure and AUD in prisoners in a correctional institution in Jimma, Southwest Ethiopia.

## Main text

### Method and materials

#### Study design and setting

We conducted a cross-sectional study in the correctional institution in Jimma, Southwest Ethiopia over a 4-week period (June–July) in 2017. In total, the facility houses 1460 prisoners (1418 men and 42 women).

#### Sample size

The sample size (n) was calculated by using the single population proportion formula, (n = $z2pq/d2$) and assuming a prevalence (P) of 50%, i.e. 0.5 (because no similar study has been performed among a prison population in Ethiopia), a 95% confidence interval (CI) (Zα/2 = 1.96), a 5% margin of error (D, 0.05), and a non-response rate of 10%. Thus, the final sample size required was 336.

#### Study procedures

We used a systematic random sampling technique to select study participants. A total of 1460 prisoners were eligible for the study. The sampling interval was 1460/336 = 4. Data were collected by five Master of Science in Psychiatry students. All data collectors were given training on the study. The five students were supervised by two Masters of Science in Public Health students and the principal investigator. A pre-test was conducted on 5% of the sample in the Agaro prison, which is located 45 km away from Jimma; on the basis of the responses in the pre-test, we corrected some ambiguous words on the data collection questionnaire. We assessed the presence of AUDs with the Alcohol Use Disorders Identification Test (AUDIT) which was developed by the World Health Organization (26). An AUDIT score ≥ 8 is considered to indicate an AUD. The sensitivity and specificity of AUDIT for AUD are 0.90 and 0.80 respectively [16] and the reliability of AUDIT in this study was 0.87 (Cronbach’s α). Adverse traumatic life events were assessed with the Life Events Checklist (LEC) (positive score if at least one traumatic event was recorded). The LEC was developed by the National Center for Posttraumatic Stress Disorder to aid in the detection of PTSD; it has been widely used in cross-cultural settings and is predictive of AUD, anxiety, depression, and PTSD [17]. We assessed nicotine dependence by the Fagerstrom Test for Nicotine Dependence (FTND) in which a score ≥ 1 indicates nicotine dependence [18]. The reliability of the FTND in this study was 0.80 (Cronbach’s α). To evaluate khat abuse we used the Drug Abuse Screening Test (DAST) a score ≥ 3 indicates abuse [19]. The reliability of the DAST in this study was 0.88 (Cronbach’s α). In addition, we used a questionnaire to assess the following variables for AUD: socioeconomic factors (age, sex, marital status, ethnicity, religion, educational status, occupation, income); environmental factors and behavioral and mental health factors. We assessed social support with the Oslo 3-item Social Support Scale [20] and psychopathy with the Psychopathy Checklist: Screening Version (PCL: SV; cutoff score ≥ 13) [21]; the sensitivity of the PCL-SV was 0.94, the specificity, 0.85, and the reliability in this study, 0.86 (Cronbach’s α).

#### Data processing and analysis

After the tests and questionnaires had been checked for completeness, EpiData Version 3.1 was used to enter the data. Then, the data were exported to the Statistical Package for Social Science version 21.0 for further analysis, and binary logistic regression analysis was used for both bivariate and multivariate analysis to explore associations and identify variables independently associated with AUD. Factors associated with the outcome variables that had a P value < 0.25 in the bivariate analysis were included in the multivariable analysis. Statistical significance was set at P < 0.05.

### Results

#### Socio-demographic characteristics

A total of 329 prisoners participated in the study. The response rate was 97.9%; 2.3% (n = 7) did not participate because they were unwilling to be interviewed about their substance use histories. Detailed information on socio-demographic characteristics is provided in Table 1. The median age of the participants was 26 years (median absolute deviation [MAD] 8.9). The majority of the participants had been residing in urban areas before imprisonment and were unmarried. The most common religion was Muslim (see Table 1).

**Table 1:** Socio-demographic characteristics of prisoners (n = 329) in the correctional institution in Jimma, Southwest Ethiopia

Continuous variables	Median	MAD	Range
Age (years)	26	8.9	16–86
Income [Birr/months]	1000	1112.0	0–35,814
Time in prison (months)	9	10.4	1–120
Duration of sentence (months)	39	46.0	0–360

Categorical variables	n	Percent
Sex		
Male	307	93.3
Female	22	6.7
Place of residence		
Urban	223	67.8
Rural	106	32.2
Religion		
Muslim	181	55.0
Orthodox	97	29.5
Protestant	38	11.6
Catholic	13	4.0
Marital status		
Single	175	53.2
Married	124	37.7
Widowed	23	7.0
Divorced/separated	7	2.1
Level of education		
Primary education	178	54.1
Secondary education	94	28.6
College and above	30	9.1
No formal education	27	8.2
Occupation		
Employed	134	40.7
Farmer	99	30.1
Student	43	13.1
Unemployed	36	10.9
Other (housewife/dressmaker)	17	5.2

#### Prevalence of lifetime trauma exposure and alcohol use disorder

Of the 329 participants, 209 (63.5%) had lifetime trauma exposure and 132 (40.1%) had AUD. Of the 209 prisoners with lifetime trauma exposure, 92 (44.0%) had AUD in the 12 months before imprisonment: 44 (21.1%) had hazardous drinking, 16 (7.7%) had harmful drinking, and 32 (15.8%) had alcohol dependence; 117 (56%) had no alcohol use problem. The most commonly encountered types of trauma in the whole group and in the prisoners with AUD were transportation accidents (whole group: n = 126, 38.3%; prisoners with AUD: n = 69, 54.8%) and physical assault (whole group: n = 91, 27.7%; prisoners with AUD: n = 64, 70.3%) (see Table 2). In the whole group, 146 (44.4%) participants had experienced two and more traumatic events. Among the prisoners with AUD (n = 119), 27 (22.7%) had no exposure to a traumatic life event, 23 (19.3%) had experienced one traumatic life event, and 69 (58%) had experienced multiple traumatic life events.

**Table 2:** Lifetime trauma exposure among prisoners (n = 329) in the correctional institution in Jimma, Southwest Ethiopia

Variables	Alcohol use disorder	
	No, n (%)	Yes, n (%)
Transportation accident (for example, car accident, boat accident, train wreck, plane crash)		
Yes	57 (45.2)	69 (54.8)
No	153 (75.4)	50 (24.6)
Physical assault (for example, being attacked, hit, slapped, kicked, beaten up)		
Yes	27 (29.7)	64 (70.3)
No	183 (76.9)	55 (23.1)
Assault with a weapon (for example, being shot, stabbed, threatened with a knife, gun, or bomb)		
Yes	32 (42.1)	44 (57.9)
No	178 (70.4)	75 (29.6)
Fire or explosion		
Yes	21 (33.9)	41 (66.1)
No	189 (70.8)	78 (29.2)
Sudden, unexpected death of someone close to you		
Yes	18 (34.0)	35 (66.0)
No	192 (69.6)	84 (30.4)
Sexual assault (rape, attempted rape, made to perform any type of sexual act through force or threat of harm)		
Yes	6 (15.0)	34 (85.0)
No	204 (70.6)	85 (29.4)
Natural disaster (for example, flood, hurricane, tornado, earthquake)		
Yes	7 (17.9)	32 (82.1)
No	203 (70.0)	87 (30.0)
Severe human suffering		
Yes	15 (35.7)	27 (64.3)
No	195 (67.9)	92 (32.1)
Sudden, violent death (for example, homicide, suicide)		
Yes	12 (31.6)	26 (68.4)
No	198 (68.0)	93 (32.0)
Life-threatening illness or injury		
Yes	16 (43.2)	21 (56.8)
No	194 (66.4)	98 (33.6)
Other unwanted or uncomfortable sexual experience		
Yes	3 (13.0)	20 (87.0)
No	207 (67.6)	99 (32.4)
Serious injury, harm, or death you caused to someone else		
Yes	12 (40.0)	18 (60.0)
No	198 (66.2)	101 (33.8)
Combat or exposure to a war zone (in the military or as a civilian)		
Yes	12 (42.9)	16 (57.1)
No	198 (65.8)	103 (34.2)
Captivity (for example, being kidnapped, abducted, held hostage, prisoner of war)		
Yes	8 (44.4)	10 (55.6)
No	202 (65.0)	109 (35.0)
Serious accident at work, home, or during recreational activity		
Yes	14 (60.9)	9 (39.1)
No	196 (64.1)	110 (35.9)
Exposure to toxic substance (for example, dangerous chemicals, radiation)		
Yes	4 (36.4)	7 (63.6)
No	206 (64.8)	112 (35.2)
Any other very stressful event or experience		
Yes	6 (25.0)	18 (75.0)
No	204 (66.9)	101 (33.1)

#### Factors associated with alcohol use disorder

Multivariable logistic regression showed that experiencing multiple traumatic life events was significantly associated with AUD: Prisoners who had experienced multiple traumatic life events were almost three times more likely to develop AUD than prisoners with no exposure to a traumatic life event (adjusted odds ratio 2.47, 95% CI 1.23, 4.94) (see Table 3).

**Table 3:** Multivariable logistic regression analysis for independent predictors of alcohol use disorder among prisoners (n = 329) in a correctional institution in Jimma, Southwest Ethiopia

Variable	Alcohol use disorder		AOR (95% CI)
	No (%)	Yes (%)	
Psychopathy			
No	200 (69.4)	88 (30.6)	Reference value
Yes	10 (24.4)	31 (75.6)	3.33 (1.25– 8.86)
Adverse traumatic life event			
No exposure to traumatic life event	93 (77.5)	27 (22.5)	Reference value
Exposure to one traumatic life event	40 (63.5)	23 (36.5)	2.04 (0.86–4.88)
Exposure to multiple traumatic life events	77 (52.7)	69 (47.3)	2.47 (1.23–4.94)
Place of residence			
Rural	89 (84.0)	17 (16.0)	Reference value
Urban	121 (54.3)	102 (45.7)	4.86 (2.38–9.94)
Khat abuse			
No	159 (83.2)	32 (16.8)	Reference value
Yes	51 (37.0)	87 (63.0)	7.39 (3.99–13.68)
Nicotine dependence			
No	186 (70.5)	78 (29.5)	Reference value
Yes	24 (36.9)	41 (63.1)	2.49 (1.16–5.34)

*AOR* adjusted odds ratio, *95% CI* 95% confidence interval

Reference value: In the analysis, this variable indicated a lower likelihood of alcohol use disorder; it was coded as zero in SPSS logistic regression

### Discussion

The present study examined the presence of trauma exposure and AUD among prisoners in a correctional institution in Jimma, Southwest Ethiopia. We found that prisoners with trauma exposure were at greater risk for AUD than prisoners with no trauma exposure: 92 (44.0%) of the prisoners with lifetime trauma exposure had an AUD in the 12 months before imprisonment, compared with 27 (22.5%) of the prisoners without lifetime trauma exposure.

Participants who had been exposed to multiple traumatic life events had higher odds of association for AUD than participants with no exposure to traumatic life events. This finding is in agreement with a study performed in Israel, which found that exposure to traumatic events was a factor for alcohol use [22]. People exposed to multiple traumatic life events are hypothesized to use alcohol to cope with negative emotional feelings, such as anger, despair, and emptiness, and to numb themselves from the experience of the traumatic event [23]. People might also use alcohol as a coping strategy to reduce awareness of the traumatic event [23].

Previous research has also provided substantial support for the self-medication hypothesis in the context of alcohol use in PTSD. Boys and Marsden found that drugs are intentionally used to suppress, control or inhibit negative affective states [8]. Furthermore, Jacobsen et al. [5] found evidence of the intentional use of alcohol to suppress typical PTSD symptoms in particular. Additionally, neuropharmacological studies have provided clear evidence that alcohol is effective in self-medicating PTSD and that withdrawal from alcohol can reactivate the symptoms of PTSD (5, 6, and 9). Waldrop et al. [7] showed that use of alcohol can help PTSD patients cope with sleep issues, depression, or intrusive memories and reduce physical hyperarousal. In the present study, the group of people who consumed alcohol was also the group with the highest trauma load. Thus, we can assume that the prevalence of PTSD was very high among these respondents and that they intentionally used substances to better deal with symptoms from the PTSD spectrum.

Exposure to traumatic events was higher among prisoners in the Jimma correctional institution, with almost two-thirds reporting having experienced a traumatic event. Although multiple exposures to traumatic events was a significant risk factor for alcohol use among prisoners, the relationship between multiple exposures to traumatic events and alcohol use is more nuanced. Although we cannot establish causality from our data, mental health professionals should recognize that multiple traumatic exposures among prisoners may be a particular risk factor for AUD. These findings also underscore the need for mental health professionals who work with prisoners who have been exposed to a variety of traumatic experiences to thoroughly assess the prisoners for AUD. Given the many barriers to help-seeking in this population, these findings suggest that treatment should target multiple psychopathologies at once. A primary health physician is thus a critical link to substance use treatment or treatment for trauma exposure for prisoners. Further research on integrated models of treatment should examine the effectiveness of such models in addressing trauma exposure and AUD among prisoners.

In summary, this study has a significant clinical impact in that it will help prison health services meet the complex mental health needs of prisoners. It demonstrates the relevance of integrated and socially acceptable care for treatment of trauma and AUDs in prisons.

### Conclusion

This study in an Ethiopian prison found a high prevalence of AUD among prisoners with lifetime trauma exposure. Prisoners with greater trauma exposure had higher odds of association for AUD than those with no trauma exposure. This study highlights that a public health intervention targeting survivors of traumatic experience needs to be designed and implemented to decrease the risk of AUD and that there is a critical need to provide treatment for prisoners with AUD.

## Limitations

- AUD were based on self-reports that the prisoners provided during an interview and therefore the prevalence of AUD may have been underestimated.
- Participants may have under reported or denied alcohol use because of social desirability bias.
- AUDIT has not yet been validated in prison populations.
- Data on mental illness were also based on self-reports, so they may be biased, and we could not verify the validity of the participants’ responses through a psychiatric interview.

## Data Availability

The datasets generated and analyzed during the current study are part of an ongoing project, and we will make them available to organizations and individuals upon official request.

## Abbreviations

AOR: adjusted odds ratio
AUD: alcohol use disorder
AUDIT: alcohol use disorders identification test
COR: crude odds ratio
DAST: drug abuse screening test
FTND: Fagerstrom Test for Nicotine Dependence
MAD: median absolute deviation
